# Inflammation as a New Therapeutic Target among Older Patients with Ischemic Heart Disease

**DOI:** 10.3390/jcm13020363

**Published:** 2024-01-09

**Authors:** Daniela Maidana, Andrea Arroyo-Álvarez, Andrea Arenas-Loriente, Guillermo Barreres-Martín, Carles Muñoz-Alfonso, Daznia Bompart Berroteran, Francisca Esteve Claramunt, Regina Blanco del Burgo, Pedro Cepas-Guillén, Sergio Garcia-Blas, Clara Bonanad

**Affiliations:** 1 INCLIVA—Instituto de Investigación Sanitaria, Biomedical Research Institute, 46010 Valencia, Spainpaqui.esteve@hotmail.com (F.E.C.);; 2 Cardiology Department, Hospital Clinic Barcelona, 08036 Barcelona, Spain; 3 Cardiology Department, Clinic University Hospital of Valencia, 46026 Valencia, Spain

**Keywords:** ischemic heart disease, older patients, inflammation, cardiovascular disease

## Abstract

Cardiovascular (CV) diseases remain a global health challenge, with ischemic heart disease (IHD) being the primary cause of both morbidity and mortality. Despite optimal pharmacological therapy, older patients with IHD exhibit an increased susceptibility to recurrent ischemic events, significantly impacting their prognosis. Inflammation is intricately linked with the aging process and plays a pivotal role in the evolution of atherosclerosis. Emerging anti-inflammatory therapies have shown promise in reducing ischemic events among high-risk populations. This review aims to explore the potential of targeted anti-inflammatory interventions in improving clinical outcomes and the quality of life for older patients with IHD.

## 1. Introduction

Cardiovascular (CV) diseases remain as a global health challenge, representing the leading cause of mortality and loss of health [1]. Among them, ischemic heart disease (IHD) is the predominant cause of morbidity and mortality [2]. It is expected that its incidence will continue to increase primarily due to the aging of the population [3]. Current research goals in the CV field focus on identifying and addressing modifiable CV risk factors to guide the development of population health strategies and establish new therapeutic targets. The critical role of inflammation in atherogenesis, plaque instability, and subsequent cardiac events is now well known. While the importance of inflammation in atherosclerosis is well established [4], its specific implications in the context of older patients with IHD remain an area of active investigation. In this population, managing IHD presents distinctive challenges, requiring a comprehensive understanding of the underlying pathophysiology along with an integral approach to balance benefits and risks due to a high burden of comorbidities and frailty [5,6]. With the increase in the aging population, it is crucial to consider new approaches to reduce the burden of CV diseases, especially IHD. This review aims to provide a comprehensive clinical overview of the relationship between aging, inflammation, and IHD. Additionally, it seeks to elucidate the potential for targeted anti-inflammatory interventions to improve the clinical outcomes and quality of life of older patients with IHD.

## 2. Inflammation and Ischemic Heart Disease

The link between inflammation, atherosclerosis, and coronary syndrome—both acute and chronic—is well known. In the 19th century, Dr. Virchow recognized the role of inflammation in atherosclerosis [7]. In recent decades, several studies have documented the association between chronic inflammatory disease association (per example, rheumatoid arthritis) and CV diseases [8]. Nowadays, there is increased interest in understanding the impact of a low-inflammatory state on the development of atherosclerosis and, ultimately, IHD.

Atherosclerosis has been defined as an ongoing inflammatory process [7]. Inflammation plays a pivotal role in the development of atherosclerosis, from its initial stages (subclinical atherosclerosis) to its progression and, ultimately, to the onset of its complications (thromboembolic events) [9]. Several hypotheses have been proposed to explain the atherosclerosis process. Among them, the oxidation hypothesis is currently the most accepted [7]. The initiation of atherosclerosis involves the accumulation of lipids (low-density lipoprotein (LDL)) in arterial walls (intima), which undergo complex oxidative modification. This process activates an immune cascade, leading to cell activation (from monocytes and lymphocytes to foam cells) and subsequent release of inflammatory cytokines such as tumor necrosis factor-alpha (TNF-α), interleukin-6 (IL-6), and interleukin-1 beta (IL-1β), as well as reactive oxygen species. These perpetuate the inflammatory response, induce vascular remodeling, and increase plaque susceptibility to rupture, leading to subsequent thrombus formation [9].

**Figure 1:**
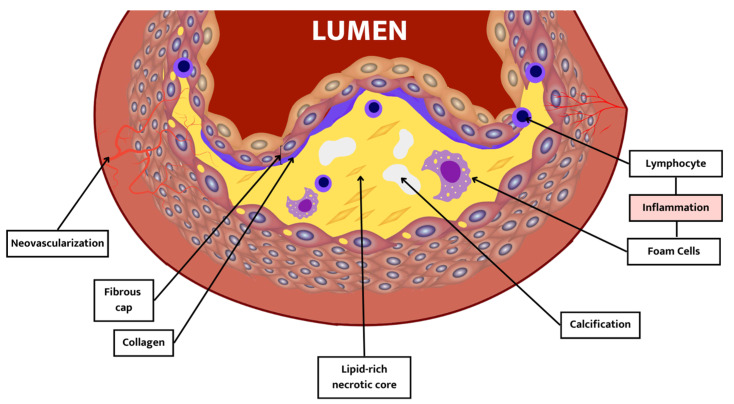
Vulnerable plaque. High-risk features of vulnerable plaque. Its presence has been associated with an increased risk of cardiovascular events.

Nevertheless, not all atherosclerotic lesions predispose people to the development of an acute CV event. This depends more on the plaque composition rather than the severity of their narrowing. Thus, we will be moving from the concept of “stable plaque” to “vulnerable plaque”, which is prone to rupture [10]. The phenotype of these vulnerable plaques, also known as thin cap fibroatheroma, is detailed in Table 1 and Figure 1.

Pathological examinations have revealed that the primary cause of myocardial infarction and cardiac death come from thrombotic events resulting from the rupture of a thin-cap fibroatheroma [11]. The two other mechanisms associated with acute events are endothelial erosion and nodular calcification. Several studies have established that plaque rupture and its subsequent healing constitute the primary mechanism driving plaque growth. Many lesions experience multiple ruptures prior to reaching a state of severe obstruction [12]. Advancements in intracoronary imaging have enabled the characterization of the morphology of coronary atherosclerotic lesions, facilitating the successful transfer of this knowledge in vivo. In the PROSPECT (A Prospective Natural History Study of Coronary Atherosclerosis) trial, approximately 700 patients with acute coronary syndromes (ACS) underwent three-vessel coronary angiography and intravascular imaging were included [13]. The objective was to determine whether subsequent major adverse cardiovascular events were related to either originally treated (culprit) lesions or untreated (non-culprit) lesions. At 3.4 years follow-up, the rate of major adverse cardiovascular events was 20.4%: 12.9% of cases were related to previous culprit lesions and 11.6% were related to non-culprit lesions. Most non-culprit lesions responsible for events were angiographically mild at baseline (±30% of stenosis) and were classified as thin-cap fibroatheromas with a large plaque burden using intracoronary imaging. Similar results were observed in a substudy of the PROMISE (Prospective Multicenter Imaging Study for Evaluation of Chest Pain) trial. In this substudy, the presence of high-risk by coronary computed tomographic angiography was present in 676 patients (15%) and carried a 70% increased risk of major adverse cardiovascular events independent of cardiovascular risk factors and obstructive coronary artery disease [14].

Significant evidence supports the association between inflammation and CV diseases [15]. Higher levels of proinflammatory cytokines (IL-1β, IL-1α, IL-6) and high-sensitivity C-reactive protein have been linked to an increased risk of major adverse cardiovascular events (MACE) [16]. In a meta-analysis of >50 prospective studies, the magnitude of risk of MACE associated with the elevation in high-sensitivity C-reactive protein resembled that observed for the elevation in total cholesterol or blood pressure [17]. As a result, the latest European Society of Cardiology Guidelines on CV diseases prevention includes low inflammation as an independent CV risk factor [18]. Recognition of the role of inflammation in IHD has led to a growing interest in the development of anti-inflammatory therapies with promising results, as will be addressed later.

## 3. Inflammation, Aging, and Ischemic Heart Disease

The aging process is marked by a gradual deterioration in physiological functions, making older patients a particularly susceptible population, with higher rates of morbidity and health loss. Notably, aging has a pronounced impact on the CV system, culminating in increased risk of developing CV diseases [19]. The contemporary approach has allowed an improvement in the prognosis of patients with IHD. However, the expected demographic shift for the coming decades represents one of the greatest challenges for healthcare systems worldwide due to an increased incidence of age-related CV disease [20]. Hence, it is imperative to identify patients in the initial stages of atherosclerosis and to modify the natural evolution of this condition (primary prevention). Alternatively, in those cases where acute events have occurred (e.g., ACS), proactive measures become essential to prevent new CV events (secondary prevention). By adopting this approach, we aim to mitigate the healthcare and socioeconomic impacts of age-related cardiovascular diseases.

In recent years, we have transitioned from a chronological aging perspective to a biological aging approach in our treatment of older patients. This approach explains better the differences which are observed between individuals of the same age. Several factors have been associated with an increased risk of aging [21]: genomic instability, telomere attrition, epigenetic alterations, loss of proteostasis, deregulated nutrient sensing, mitochondrial dysfunction, cellular senescence, stem cell exhaustion, and altered intercellular communication. In most cases, inflammation plays a central role. The interplay among this triangle—inflammation, aging, and cardiovascular disease—has derived to the conceptualization of “Inflamm-ageing” [22,23].

Inflamm-ageing is characterized as chronic, low-grade, systemic inflammation that accompanies aging in the absence of other factors (i.e., chronic infection). This low-grade inflammation significantly impacts the CV system and may explain the increased risk of CV events among older patients [23]. Recognizing its significance, current research is toward enhancing our comprehension of the underlying mechanisms driving this chronic inflammation associated with aging. Several (pre-) clinical studies have identified different processes involved in inflamm-ageing: cellular senescence, immunosenescence, garbaging, microbiote/nutrition, and clonal hematopoiesis of an indeterminate potential epigenetic/genetic clock. The main characteristics of this process are included in Table 2 [22].

Consequently, aging and chronic low-grade inflammation converge to augment the risk of CV diseases, especially IHD. As mentioned earlier, the processes associated with inflamm-ageing not only promote atherosclerosis, but also modulate traditional CV risk factors [23]. Conditions such as obesity, hypertension, and diabetes mellitus create a favorable environment for inflamm-ageing. This correlation creates a vicious cycle, where CV risk factors fuel chronic systemic inflammation, and in turn, inflammation exacerbates the deleterious effects of these risk factors.

Chronic iron deficiency exemplifies this vicious cycle. Iron deficiency is prevalent among older patients, affecting around 20% at age 80 [28], rising to 60% in cases with concomitant IHD [29]. Its presence has been associated with an increased risk of CV diseases, especially IHD [30]. Two physiopathological processes explain the onset of iron deficiency: (1) insufficient intake/absorption or chronic blood loss leading to an iron storage deficit; (2) reduction in circulating iron (functional iron deficiency). In the latter, chronic inflammation significantly contributes to the initiation and continuation of this condition. Hepcidin, an acute protein expressed in the liver, plays a crucial role as a negative regulator. Elevated levels of hepcidin are linked to a reduction in both iron absorption and mobilization. Hepcidin regulates iron homeostasis by modulating the absorption of iron from the small intestine into the bloodstream (through ferroportin) and by regulating iron release from macrophages in the spleen and the liver [31]. Therefore, chronic inflammation among older patients (inflamm-ageing) increases hepcidin level, subsequently reducing iron absorption and mobilization from the reticuloendothelial system [32]. In a recent study including 12,000 individuals from three large European registries, iron deficiency was associated with a 24% increased risk of IHD and 26% increased risk of CV mortality [31]. Thus, the study highlighted the association with a higher incidence of coronary disease and its greater prevalence among older individuals.

Following the previous, older patients with higher levels of inflammatory biomarkers are at an enhanced risk of developing IHD and its acute complications. However, due to the involvement of diverse pathways in the inflamm-ageing process, reversing this situation by targeting a single pathway is not feasible; instead, an integrated approach is necessary. This becomes even more crucial for older patients with IHD, given their clinical complexity and burden of comorbidities. Hence, inflamm-ageing should be considered a syndrome characterized by several disorders involving different systems. Therefore, understanding the role of inflamm-ageing in clinical settings is crucial or the development of novel health prevention strategies and effective therapeutic interventions tailored for this population.

## 4. Anti-Inflammation Strategies in Ischemic Heart Disease

The role of inflammation in atherosclerosis and IHD has garnered increased attention in recent years, given the latest research revealing a complex interplay between this process and cardiovascular pathophysiology, as previously described. The acknowledgment of inflammation’s role has prompted researchers and clinicians to explore targeted interventions aimed at mitigating the inflammatory cascade associated with IHD. Consequently, recent trials have tested the hypothesis that anti-inflammatory drugs can reduce the incidence of MACE in patients with IHD [33,34].

We will comprehensively examine different anti-inflammatory strategies utilized in the context of IHD and their outcomes, specifically emphasizing their potential impact on older patients. Hence, we will include traditional pharmacological approaches to emerging interventions, including the role of lifestyle modifications and dietary interventions.

### 4.1. Pharmacological Anti-Inflammatory Therapy

#### 4.1.1. Traditional Pharmacological Anti-Inflammatory Therapy

The benefits of low-dose aspirin (100 mg/day) in the secondary prevention of MACE in IHD patients is well documented, although the mechanisms of this effect are unclear [35]. The two primary hypotheses that could explain its benefits are its antiplatelet and anti-inflammatory effects, with the former being better understood than the latter. Nevertheless, several clinical studies have demonstrated that the administration of low-dose aspirin is associated with a decrease in proinflammatory biomarkers such as IL-6, IL-1, and C-reactive protein among patients IHD [36,37]. Moreover, a sub-analysis from The Physicians’ Health Study showed the reduction associated with the use of aspirin in the risk of a first myocardial infarction appears to be directly related to the level of C-reactive protein [38]. In this study, healthy participants were randomly assigned to receive aspirin or placebo. The level of CRP was measured at the beginning of the study. Patients in the quartile with the highest CRP values had higher risk of myocardial infarction or stoke compared to those in the lowest quartile. The use of aspirin was associated with significant reductions in those participants in the highest quartile (55% of reduction) but with only small, nonsignificant reductions in those in the lowest quartile (13.9% of reduction). This study demonstrated that the greater the risk patients face, the greater the benefits of using aspirin in CV prevention, with inflammation (measured through CRP) playing a central role in the pathogenesis of atherosclerosis. This approach, detecting high-risk patiens, is supported by the results of ASPREE trial where the daily use of aspirin did not provide a benefit with regard to the primary end point of disability-free survival among healthy older adults [39].

On the other hand, the benefits of lipid lower therapy such as statins or novel PCSK9 inhibitors extends beyond the isolated reduction in LDL levels. The pleiotropic effect of statins in the context of IHD is widely documented with evidence showing that statins could have favorable and clinically relevant anti-inflammatory effects independent of lipid lowering [33]. Data from the CARE (Cholesterol and Recurrent Events) [40] and PRINCE (Effect of statin therapy on C-reactive protein levels: the pravastatin inflammation/CRP evaluation) [41] trials showed that the administration of pravastatin reduced C-reactive protein levels in a largely LDL-C-independent manner. This effect was observed in both primary (PRINCE trial) and secondary prevention (CARE trial). Similar results were observed in PROVE IT–TIMI 22 (Pravastatin or Atorvastatin Evaluation and Infection Therapy—Thrombolysis In Myocardial Infarction 22) study, which suggested dual mechanisms of benefit of statin therapy: LDL-lowering, and a direct anti-inflammatory effect independent of LDL-lowering. These were reflected by a reduction in high-sensitivity CRP [42]. The JUPITER (Justification for the Use of Statins in Prevention: an Intervention Trial Evaluating Rosuvastatin) trial went further and evaluated the role of statins (rosuvastatin 20 mg. vs. placebo) in patients without previous CV disease with LDL-C levels below 130 mg/dL, but with high-sensitivity CRP levels ≥ 2 mg/L [43]. The JUPITER trial was stopped early because of greater reduction in the primary end point of MACE: 54% of reduction in myocardial infarction, 48% in stroke, 46% in the need for arterial revascularization, and 20% in all-cause mortality. The number of patients to treat (NNT) was 32 for MACE (myocardial infarction, stroke, and death), showing the great benefits of statins in patients with a proinflammatory state beyond LDL levels.

Given the important role of inflammation in the CV setting, this effect of statins might explain, in addition to LDL reduction, their reduction in MACE. On the other hand, recent studies have demonstrated the capability of the new PCSK9 inhibitors to reduce atherosclerotic burden by regressing vulnerable plaques in patients following ACS [44,45,46] and patients with familiar hypercholesterolemia [47]. It is important to emphasize that the notable reduction in MACE observed in the pivotal trials of these pharmaceutical families primarily arises from their capacity to decrease LDL levels. However, further exploration is required to fully elucidate the extent and significance of their anti-inflammatory effects [48].

#### 4.1.2. Novel Pharmacological Anti-Inflammatory Therapy

##### Canakinumab

The Canakinumab Anti-inflammatory Thrombosis Outcome Study (CANTOS) was a randomized, double-blind, placebo-controlled trial involving stable patients with previous myocardial infarction; this study evaluated whether canakinumab could prevent recurrent vascular events among those who have a persistent proinflammatory response (high-sensitivity CRP ≥ 2 mg/L) [49]. Canakinumal is a fully human monoclonal antibody targeting interleukin-1β. As previously cited, interleukin-1β has been involved in the proinflammatory cascade leading to atherosclerosis [11]. This was the first study to evaluate the role of a direct anti-inflammatory therapy in cardiovascular events. A total of 10,061 patients were included and were randomly assigned in a 1:1:1 ratio to receive placebo, canakinumab at a dose of 150 mg, or canakinumab at a dose of 300 mg. At a median follow-up of 3.7 years, the incidence rate for the primary end point (nonfatal myocardial infarction, nonfatal stroke, or cardiovascular death) was 4.50 events per 100 person-years in the placebo group, 3.86 events per 100 person-years in the 150-mg group, and 3.90 events per 100 person-years in the 300-mg group—these differences were statistically significant. No difference was observed in all-cause mortality between canakinumab and placebo groups. In the canakinumab groups, a higher percentage of fatal infections/sepsis was observed than in the placebo group (incidence rate, 0.31 vs. 0.18 events per 100 person-years; *p* = 0.02). On the other hand, cancer mortality was significantly lower with canakinumab than with placebo.

Regarding the role of this therapy among older patients, a post hoc analysis of the CANTOS trial evaluated the effect of canakinumab in frailty [50]. In a total of 9942 participants’ baseline frailty index were calculated. Of those, 13% of participants were classified as frail. In this sub-analysis, the primary findings of CANTOS trial in terms of canakinumab-associated cardiovascular event reduction were unchanged in analyses stratified by baseline frailty, showing the effective and safety of this therapy in frail patients. It is notable that a higher percentage of fatal sepsis was detected among older patients with diabetes, indicating that neutropenia should be monitored among those patients under this therapy to prevent infections.

##### Colchicine

Colchicine is an immunomodulator that suppresses inflammation by inhibiting neutrophil migration and degranulation and promotes collagen degradation by stimulating collagenase activity [51]. Its use is approved in different inflammatory diseases such as gout, familiar Mediterranean fever, and pericarditis, among others. In recent years, there has been an increasing interest in evaluating its role in CV disease, especially in IHD. Several reasons make colchicine an attractive molecule to evaluate its anti-inflammatory effect in patients with IHD: wide availability, low cost, and tolerable side-effect profile. Thus, Colchicine has emerged as a novel CV prevention treatment. Several clinical trials have evaluated the effectiveness and safety of colchicine among patients with different types of IHD (acute and chronic coronary syndrome). A summary of the main results of these studies is presented in Table 3 [52,53,54,55,56,57].

In a prespecified analysis included in the LoDoCo2 (Low-Dose Colchicine for Secondary Prevention of Cardiovascular Disease) trial [52], patients aged above 65 years showed greater benefits with colchicine compared to those below 65 years. No data regarding this subgroup of patients are available in the rest of the studies. These studies have contributed valuable insights into the potential benefits of colchicine in managing cardiovascular conditions, particularly in reducing adverse events and inflammation in patients with IHD. The last European Society of Cardiology on CV prevention in clinical practice publication suggested that high-risk CV disease patients could benefit from treatment with colchicine (0.5 mg daily) as secondary prevention if other risk factors are not controlled [18]. However, these results should be used with caution. Adverse effects (digestive intolerance, myalgias, and pneumonia) associated with the use of colchicine are not negligible. Considering the higher burden of associated comorbidities among older patients, its benefits and risks should be evaluated individually for each patient.

##### Methotrexate

After the CANTOS trial, the research in this field moved to find other drugs with a similar anti-inflammatory profile which might be inexpensive and more available. Low-dose methotrexate was proposed and evaluated in the CIRT (Cardiovascular Inflammation Reduction Trial) [58]. This was a randomized, double-blind, placebo-controlled, investigator-initiated trial that included patients with previous history of IHD and type 2 diabetes mellitus or the metabolic syndrome. The participants were randomly assigned to receive either low-dose methotrexate (with an initial dose of 15 mg) or a placebo. A total of 4786 participants were finally included in the study. After a median follow-up of 2.3 years, first occurrence after randomization of a final primary end-point event (nonfatal myocardial infarction, nonfatal stroke, cardiovascular death, or hospitalization for unstable angina that led to urgent revascularization) was reported in 201 patients in the methotrexate group and in 207 in the placebo group (incidence rate, 4.13 vs. 4.31 per 100 person-years; hazard ratio, 0.96; 95% confidence interval [CI], 0.79 to 1.16; *p* = 0.67). There were no differences in any other prespecified CV end point or in any individual component of these end points. Rates of serious adverse events, including bleeding and infection, were similar in the two groups. Thus, low-dose methotrexate failed to decrease CV events at the follow-up in patients with IHD. Moreover, the administration of low-dose methotrexate did not reduce levels of interleukin-1β, interleukin-6, or CRP.

##### Ziltivekimab

Ziltivekimab, a novel IL-6 ligand inhibitor, has emerged as a potential therapeutic option for atherosclerotic disease. IL-6, a key cytokine in innate immunity, plays a pivotal role in the NLRP3 inflammasome pathway, linking to IL-1 and IL-6 and ultimately leading to the production of the inflammatory biomarker CRP (10). Ziltivekimab aims to disrupt these inflammatory pathways, particularly in the context of cardiovascular diseases. In the RESCUE trial, a randomized, double-blind, placebo-controlled phase 2 study, ziltivekimab demonstrated significant efficacy in reducing biomarkers of inflammation and thrombosis, including high-sensitivity CRP, fibrinogen, serum amyloid A, haptoglobin, secretory phospholipase A2, and lipoprotein (a). The trial, conducted among individuals with chronic kidney disease and elevated CRP, showed dose-dependent reductions in these biomarkers over 24 weeks, with no significant adverse effects observed [59]. Importantly, the reduction in high-sensitivity CRP was notably larger than that achieved in the CANTOS trial using an upstream IL-1β inhibitor. These promising results have paved the way for the ZEUS trial. This large-scale cardiovascular outcome study aims to investigate the impact of ziltivekimab in patients with chronic kidney disease, elevated high-sensitivity CRP, and established cardiovascular disease. If confirmed in larger long-term trials, the safety and efficacy profile of ziltivekimab may position it uniquely among commercially available IL-6 inhibitors, offering a potential breakthrough in addressing residual inflammatory risk in atherosclerotic cardiovascular disease [60].

##### Others

Several other anti-inflammatory drugs have undergone clinical trials to assess their potential in preventing CV events in high-risk patients. However, none have succeeded in demonstrating significant CV benefits. Table 4 provides a summary of these trials [23,61,62,63,64,65].

### 4.2. Lifestyle Modifications and Dietary Interventions

Not only have pharmacological therapies demonstrated efficacy in reducing inflammation, but lifestyle modifications and dietary interventions have also shown considerable promise [66,67]. These interventions hold substantial potential in mitigating the risks associated with IHD, and their benefits extend notably to older patients, positively impacting other associated conditions.

A sedentary lifestyle significantly contributes to several CV risk factors, such as type 2 diabetes mellitus, arterial hypertension, and metabolic syndrome, among others. The accumulation of visceral fat associated with overweight and obesity has been proposed as a proinflammatory source, leading to activation of inflammatory pathways though adipokines [68]. The prevalence of overweight/obesity continues to rise worldwide, and older people are also affected [69]. Among older adults, obesity not only impacts the CV system but also is associated with a higher prevalence of cancers, osteoarticular diseases, dementia, and depression [70]. It is mandatory for health professionals to be aware of this disease and fight to overcome its fatal complications.

Engaging in regular physical activity has proven to be a potent anti-inflammatory tool. Several mechanisms might explain the anti-inflammatory effect of exercise, such as the reduction in visceral fat mass and the increased production and release of anti-inflammatory cytokines from contracting skeletal muscle [71]. A recent metanalysis involving 1250 participants evaluated the effect of aerobic exercise on inflammatory markers in middle-aged and older adults [72]. Aerobic exercise had a positive effect on reduction in CRP, TNF-α, and IL-6 among older participants. On the other hand, a simple activity like walking has a positive impact on CV health. A recent study found that walking at least 3867 steps a day started to reduce the risk of dying from any cause, and 2337 steps a day reduced the risk of dying from CV diseases [73]. The more you walk, the greater the health benefits: an increase of 500 steps a day was associated with a 7% reduction in dying from cardiovascular disease. The positive effects of exercise are not only explained by a reduction in systemic inflammation but also by improvements in blood lipid profiles, endothelial function, and myocardial regeneration [71]. Thus, encouraging older patients with IHD to adopt a tailored exercise regimen, combining aerobic activities, resistance training, and flexibility exercises, can significantly improve overall CV health. Cardiac rehabilitation plays a central role in addressing this question. Evidence from randomized controlled trials and meta-analyses supports the efficacy of cardiac rehabilitation on clinically relevant outcomes such as reduced long-term morbidity and mortality [74]. However, older patients are frequently under-referred to cardiac rehabilitation due to clinical, social, or cultural barriers. This fact occurs despite evidence demonstrating the effectiveness of cardiac rehabilitation in the elderly [75,76,77].

Nevertheless, cardiac rehabilitation moves further exercise therapy, integrating other lifestyle modifications, such as dietary and stress management. Diets wield immense influence over individuals’ inflammatory status [66]. A Mediterranean-style diet that is rich in fruits, vegetables, whole grains, fish, and healthy fats (such as olive oil and nuts) has exhibited anti-inflammatory properties [78] and it has been associated with CV benefits in both primary and secondary prevention [79,80]. The PREDIMED (Prevención con Dieta Mediterránea) trial showed that the incidence of major cardiovascular events was lower among those assigned to a Mediterranean diet supplemented with extra-virgin olive oil or nuts than among those assigned to a reduced-fat diet [79]. Similar results were observed in the CORDIOPREV (Coronary Diet Intervention with Olive oil and Cardiovascular Prevention study) trial in secondary prevention [80]. Encouraging older adults with IHD to embrace a Mediterranean diet can effectively lower inflammatory markers, reduce the risk of recurrent cardiac events, and improve long-term prognosis. Moreover, stress management techniques and sufficient sleep are integral components of lifestyle modifications that impact inflammation in IHD [81,82]. Chronic stress contributes to systemic inflammation, exacerbating CV risk [83]. Implementing stress-reduction techniques like mindfulness meditation, yoga, or deep breathing exercises can mitigate stress-induced inflammation [84,85].

## 5. Clinical Implications and Future Directions

Traditional management strategies for preventing atherosclerosis and its complications, notably IHD, have primarily emphasized the modification of classic risk factors, such as LDL levels. However, despite the latest efforts and unprecedented advances in the treatment of IHD, a considerable residual risk of CV events persists, highlighting the necessity of research into novel CV risk factors [86]. This need is even more important among older patients due to the increased incidence of IHD in this population subgroup and the demographic trend towards an aging population. In our field, it is estimated that the life expectancy of women will exceed to 90 years in the coming decades, an existing reality in daily practice [6,20,87,88]. All these factors underscore the imperative need to identify new therapeutic targets aimed at reducing the progression of atherosclerosis and its associated cardiac complications. Considering the significant role of inflammation in both the pathogenesis and progression of IHD, it represents an optimal target for mitigating the burden of CV disease.

As stated previously, there is a strong association between aging and inflammation. Therefore, anti-inflammatory treatments could reduce cardiovascular events and specifically their cardiac complications among older patients. Nevertheless, it is crucial to increase investigations into medications with acceptable costs, wide accessibility, and well-tolerated side effects, to attain an optimal cost/effectiveness ratio. CANTOS trial showed the positive effect of canakinumab for secondary prevention in patients with previous IHD [49]. However, a cost-effectiveness analysis demonstrated canakinumab is not cost-effective at its current market price: it was estimated that an increase in quality-adjusted life-years of 0.13 and an increase in costs of USD 832,000 yields an incremental cost/effectiveness ratio of USD 6.4 million per QALY gained [89]. The results associated with the use of colchicine are different. A metanalysis that included 11,816 patients demonstrated that low-dose colchicine reduced the risk of MACE as well as that of myocardial infarction, stroke, and the need for coronary revascularization among a broad spectrum of patients with coronary disease. The cost-effectiveness analyses of COLCOT trial [90] indicated that the addition of colchicine to standard-of-care therapy after ACS is economically dominant and therefore generates cost savings: lifetime per-patient costs were reduced by 69% and quality-adjusted life years increased with colchicine therapy from 8.82 to 11.68. Of note, low-dose colchicine is contraindicated in patients with significant renal or liver dysfunction and should be temporarily discontinued when taking concomitant agents such as clarithromycin, ketoconazole, and cyclosporine that share metabolism pathways. Some concerns regarding its safety profile have emerged due to notable occurrences of digestive intolerance, myalgias, and an increased susceptibility to pneumonia; thus, these factors should be considered among older patients with frailty and high burden of comorbidities [91]. The CLEAR SYNERGY (Organization to Assess Strategies for Ischemic Syndromes OASIS 9) trial will provide more information about the role of colchicine; this study includes 6440 participants after ACS (NCT03048825).

**Figure 2:**
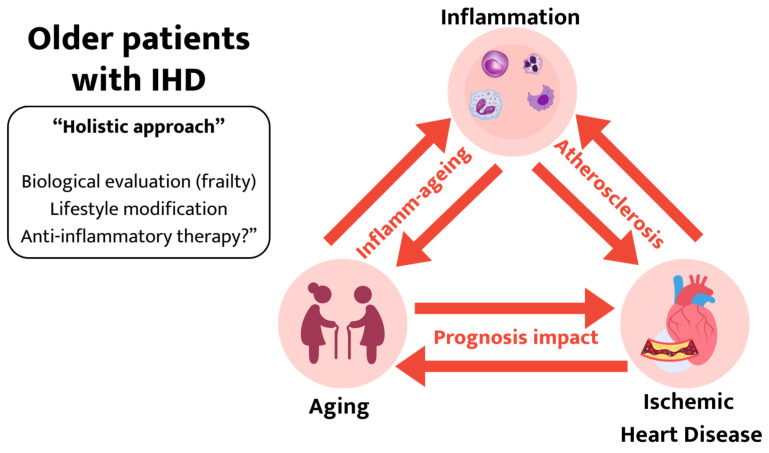
Holistic approach among older patients with ischemic heart disease. A strong correlation among inflammation, aging, and CV disease has been described. Holistic approaches among older patients with IHD are recommended to improve their prognoses.

Older patients face unique challenges due to the complexity and frequency of comorbidities. An integrated intervention tailored to these individuals should involve different strategies, addressing not only the CV condition but also the associated health issues and frailty. Accordingly, this holistic approach involves lifestyle modifications, including tailored exercise programs and dietary adjustments, along with pharmacological interventions (Figure 2). Most clinical trials targeting specific anti-inflammatory downstream targets failed to meet their primary endpoints with respect to a decrease in CV events [92]. Nevertheless, a range of opportunities has emerged for the exploration of novel anti-inflammatory treatments targeting inflammation to prevent atherosclerosis. However, it is evident that further research is essential if we are to fully comprehend the roles of anti-inflammatory and immunomodulatory interventions into atherosclerotic CV disease. A more comprehensive understanding of the intricate components involved in the inflammatory process, including its relationship with aging, is essential. This knowledge should be tailored to include the necessary holistic approaches when managing older patients with IHD. As demonstrated in this review, lipid lowering and inflammation inhibition are not in conflict but are synergistic [93]. In the future, combined use of aggressive LDL-C-lowering and inflammation-inhibiting therapies may become the standard of care for most atherosclerosis patients [94], including older patients.

## 6. Conclusions

In conclusion, the evidence shows a strong correlation among inflammation, aging, and CV disease. Older patients with IHD are more prone to recurrent cardiovascular events that will impact their prognoses. The role of anti-inflammatory therapy could diminish recurrent events and enhance quality of life. Nonetheless, additional studies targeting this high-risk population are imperative if we are to ascertain its actual impact.

## Figures and Tables

**Table 1:** Vulnerable plaque features [10].

Component	Description
Thin fibrous cap	Cap thickness < 65 μm. It occurs due to an imbalance between collagen synthesis and breakdown.
Lipid-rich necrotic core	A lipid-rich atheromatous core: hypocellular, low grade of supporting collagen, with high free cholesterol content in the core.
Inflammation	Inflammation is mainly distributed under the fibrous cap, not in the plaque core.
Calcification	Vulnerable plaques are less calcified than stable plaques.
Neovascularization	Higher micro-vessel density in vulnerable plaque than in stable plaque.

**Table 2:** Main elements contributing to inflamm-ageing.

Element	Characteristics
Cellular Senescence	- Cellular senescence is defined as a permanent proliferation arrest [24]. - Aging is associated with an increasement of senescence cells (SC). - SC are accumulated in CV system → ↑ reactive oxygen species and proinflammatory cytokines → chronic inflammation.
Immunosenescence	- Immunosenescence is a multifactorial process that provokes age-dependent functional changes in immune cells. - This dysregulation of innate and adaptive immune cells can promote age-related changes in CV function.
Garbaging	- It is proposed that aging is associated with an increasement of “garbage” production (cellular debris and misplaced proteins) [25]. - This waste materials lead to a release of inflammatory mediators, increasing inflammation and, finally, CV disorders.
Microbiome	- The composition of the intestinal microbiome changes with aging. With aging, there is an increase in anaerobes species in the intestinal microbiome. Their increase has been associated with higher levels of proinflammatory cytokines such as IL-6 and IL-8. - Aging is associated with a reduction in mucosal barrier function, increasing the absorption of proinflammatory components [26].
Clonal Hematopoiesis of Indeterminate Potential (CHIP)	- CHIP → presence of an expanded somatic blood–cell clone in persons without other hematologic abnormalities. CHIP is more frequent with aging due to somatic mutations accumulate with age. - The presence of CHIP in peripherical-blood cells has been recognized as a strong predictor of CV events (×2 risk of IHD) [27].
Epigenetic/genetic clock	- Aging + environmental stimuli → large modifications in epigenetic patterns. - This epigenetic change includes: (a) Loss of heterochromatin. (b) Modification of epigenetic markers at specific genomic locations. (c) Increased epigenetic variability.

**Table 3:** Main elements contributing to inflamm-ageing [52,53,54,55,56,57].

Study	Population	Control	Main Results
LoDoCo trial	CCS n = 532	Conventional treatment	Follow-up 3 years Primary endpoint (ACS, cardiac arrest, stroke): 5.3% vs. 16%; *p* < 0.001 GI intolerance (2.5%) in Colchicine group
LoDoCo2 trial	CCS n = 5522	Placebo	Follow-up 28.5 months Primary endpoint (CV death, MI, stroke, ischemia-driven coronary revascularization): 6.8% vs. 9.6%; *p* < 0.001 Trend to ↑ rate of non-CV death (1.9%; HR, 1.51 [95% CI, 0.99–2.31]) Myalgia 21.2% and GI intolerance 15.4%
COPS trial	ACS n = 795	Placebo	Follow-up 12 years Primary endpoint (death, ACS, stroke, ischemia-driven coronary revascularization): 6.1% vs. 9.5%; *p* = 0.09 Significant higher rate of non-cardiovascular death (5 vs. 0; *p* = 0.024)
COLCOT trial	ACS n = 4745	Placebo	Follow-up 22.6 months Primary endpoint (CV death, ACS, cardiac arrest, stroke, coronary revascularization): 5.5% vs. 7.1%; *p* = 0.02 Significant higher rate of Pneumonia (0.9% vs. 0.4%; *p* = 0.03)

CCS = chronic coronary syndrome; ACS = acute coronary syndrome; GI = gastrointestinal; CV = cardiovascular; MI = myocardial infarction. A colchicine dose of 0.5 mg/24 h was used in all studies.

**Table 4:** Summary of failed trials with other anti-inflammatory therapies [23,61,62,63,64,65].

Study	Drug	Population	Main Results
ARISE	Succinobucol	ACS n = 6144	Failure to reduce fatal and non-fatal cardiovascular events in patients with recent ACS.
MRC-ILA Heart	Anakinra	ACS n = 182	Reduction in inflammatory markers 14 days after ACS. Excess of MACE events 1 year after the treatment.
SOLID-TIMI-52	Darapladib	ACS n = 13,026	Failure to reduce major coronary events in patients with recent ACS.
STABILITY	Darapladib	CCS n = 15,828	Failure to reduce fatal and non-fatal cardiovascular events in patients with CCS.

CCS = chronic coronary syndrome; ACS = acute coronary syndrome; GI = gastrointestinal; CV = cardiovascular; MI = myocardial infarction. A colchicine dose of 0.5 mg/24 h was used in all studies.

## Data Availability

Not applicable.
